# Protocol for quantitative analysis of RNA 3′-end processing induced by disassociated subunits using chromatin-associated RNA-seq data

**DOI:** 10.1016/j.xpro.2023.102356

**Published:** 2023-06-15

**Authors:** Jie Huang, Lijun Bao, Junyi Zhu, Xiong Ji

**Affiliations:** 1 Key Laboratory of Cell Proliferation and Differentiation of the Ministry of Education, School of Life Sciences, Peking-Tsinghua Center for Life Sciences, Peking University, Beijing 100871, China

**Keywords:** Bioinformatics, Genomics, Gene Expression

## Abstract

Sequencing chromatin-associated RNA using libraries from the chromatin fraction makes it possible to characterize RNA processing driven by disassociated subunits. Here, we present an experimental strategy and computational pipeline for processing chromatin-associated RNA-seq data to detect and quantify readthrough transcripts. We describe steps for constructing degron mouse embryonic stem cells, detecting readthrough genes, data processing, and data analysis. This protocol can be adapted to various biological scenarios and other types of nascent RNA-seq, such as TT-seq.

For complete details on the use and execution of this protocol, please refer to Li et al. (2023).^1^

## Highlights

• Development of the SDDS system to study dRPB3
• De novo identification of readthrough transcripts genome-wide
• Quantification of readthrough indices based on chromatin-associated RNA-seq
• Pipeline for comparing 3′-end processing defects upon RPB3 and dRPB3 depletion

## Abstract (editor highlights)

Publisher’s note: Undertaking any experimental protocol requires adherence to local institutional guidelines for laboratory safety and ethics.

## Graphical abstract

**Figure:**
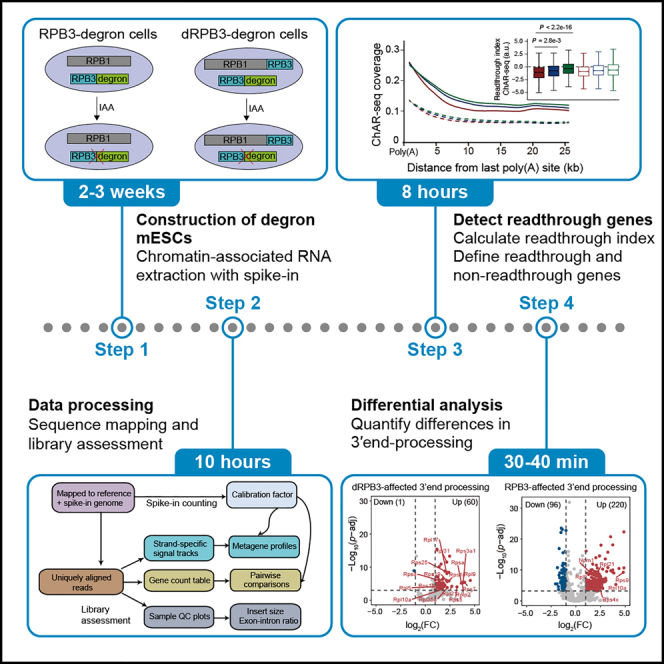

## Before you begin

This section includes the minimal hardware requirements, the installation procedures of essential tools, as well as the construction of RPB3 and dRPB3 degron systems in mouse embryonic stem cells (mESCs), which are the fundamental cell lines used for creating ChAR-seq libraries and subsequent sequencing.

### Construction of RPB3 and dRPB3 degron mESCs

**Timing: ∼6–7 weeks**

**Figure 1:**
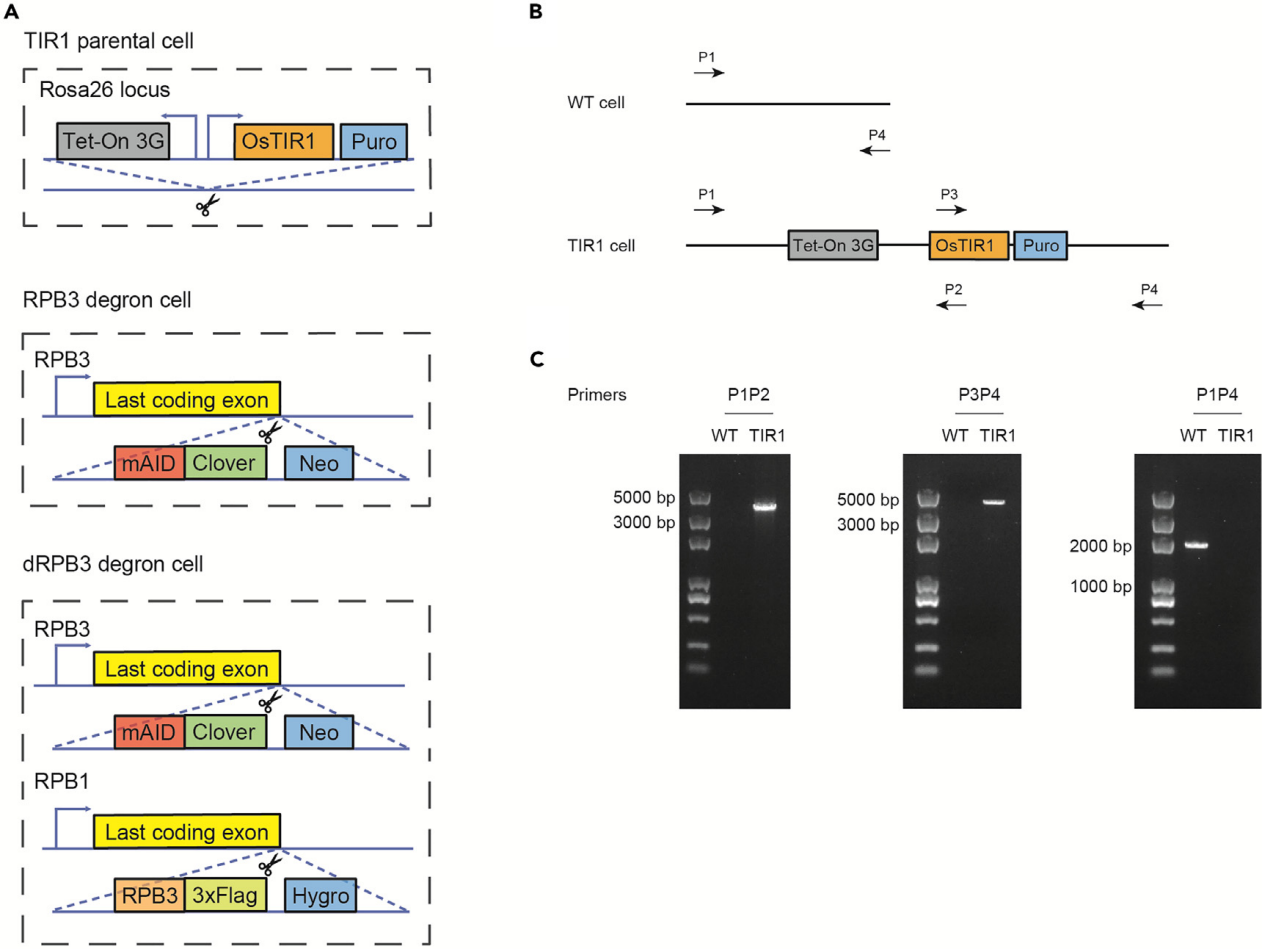
Experimental scheme used to construct TIR1, RPB3 degron, dRPB3 degron cells and parental mESCs expressing OsTIR1 (A) tet-OsTIR1 is inserted into Rosa26 locus with CRISPR/Cas9 and selected with 5 μg/mL puromycin. Middle: mAID-GFP tag is inserted at the last exon of RPB3 gene with CRISPR/Cas9 and selected with 500 μg/mL geneticin for one week. Bottom: mouse cDNA of RPB3 with 3xFlag tag is inserted at the last exon of RPB1 gene with CRISPR/Cas9 and selected with 250 μg/mL hygromycin for one week. (B) A schematic illustration of genotyping primers used to detect the genotype of TIR1 parental cells at Rosa26 locus. (C) Genotyping results to detect the homogeneity of TIR1 parental cells. The product length of primers P1P2, P3P4, and P1P4 is 3504 bp and 3841 bp, respectively. Since the PCR product of P1P4 in TIR1 parental cells should be longer than 6000 bp, it is not easy to extract such long genomic DNA from cells. Therefore, we just got a product with 1827 bp of P1P4 that can be derived from WT cells but no bands in TIR1 parental cells.

***Note:*** The mES cells were cultured in DMEM-KO (Thermo Fisher, Cat#10829018), supplemented with 15% FBS (Gibco, Cat#10099141C), 1× penicillin/streptomycin (Gibco, Cat#15140122), 1 × L-glutamine (Gibco, Cat#25030081), 1 × non-essential amino acids (Gibco, Cat#11140050), 1× nucleoside (Millipore, Cat#ES-008-D), 3 μM CHIR99021 (Selleck, Cat#S1263), 1 μM PD0325901 (Selleck, Cat#S1036), 143 μM 2-mercaptoethanol (Thermo Fisher, Cat#M3148), and maintained at 37°C cell incubators with 5% CO2. The Schneider 2(S2) cells were cultured in complete Schneider's Drosophila Medium (Invitrogen, Cat#21720024) supplemented with 10% FBS (CellMax, Cat#SA311.02) at room temperature.

1. Use RPB3 C-terminal targeting sgRNA, CRISPR/Cas9, and donor plasmid to generate RPB3 degron cell line (Figure 1).
  a. Co-transfect RPB3 C-terminal targeting sgRNA, CRISPR/Cas9, and donor plasmids into *Os*Tir parental cell.
  b. Co-transfect mESCs using FuGene HD transfection reagent, following the manufacturer’s instructions.
  c. After two days, treat the cells with 500 μg/mL Geneticin for one week to select successfully transfected cells, change the medium every two to three days.
  d. Pick colonies and culture in a 48-well plate for genotyping.
  e. Design forward and reverse genotyping primers for the region about 200 bp upstream or downstream from the sgRNA targeting sites.
  f. Amplify genomic DNA by PCR and check for successful knock-in by observing PCR products around 3.5 kb larger than those from wild-type cells.
  g. Sanger sequencing the PCR-amplified genomic DNA products to verify the homogeneous knock-in.
  h. Verify correct expression of RPB3 fused with mAID-GFP tag by Western blotting.

2. Use RPB1 C-terminal targeting sgRNA, CRISPR/Cas9, RPB1-linker-RPB3 donor plasmid and RPB3 degron cells to generate dRPB3 degron cell line (Figure 1).
  a. Plate RPB3 degron cells to achieve 50%–70% confluency.
  b. Transfect RPB1 C-terminal targeting sgRNA, CRISPR/Cas9, and RPB1-linker-RPB3 donor plasmids into the cells.
  c. Incubate the cells at 37°C in a humidified atmosphere containing 5% CO_2_ for two days.
  d. Add hygromycin B (250 μg/mL) to the culture medium and incubate the cells for one week, changing the medium every two to three days.
  e. Pick colonies and culture in a 48-well plate for genotyping.
  f. Amplify the genomic DNA of the dRPB3 degron cells by using genotyping primers for RPB1-CTD degron cells.
  g. Analyze the genotyping results to confirm the successful generation of dRPB3 degron cells by Sanger sequencing and Western blotting.

***Note:*** All primer sequences were listed in key resources table.

### Preparation of the hardware and computing environment

**Timing: ∼3–4 h**

#### Hardware

This bioinformatic pipeline is designed to be run in the command line under UNIX operating systems (e.g., Linux or macOS). A multicore computer with at least 16 cores and 64 GB RAM is suggested, but needed computing resources depend heavily on the size of the analyzed dataset.

#### Software environment

Anaconda for Python (version 3.7.4 is recommended) can be downloaded from https://www.anaconda.com.

R can be downloaded from https://cran.r-project.org/.

Perl can be downloaded from https://www.perl.org/.

The RStudio integrated development environment (IDE) that provides a graphical interface to R can be downloaded from https://www.rstudio.com/products/rstudio/.

### Installation of the required software

**Timing: ∼1 h**

3. Add channels to Conda configuration and install required software packages.

> conda config --add channels defaults

> conda config --add channels bioconda

> conda install -c bioconda bowtie2 sambamba deeptools rseqc cutadapt wiggletools bedtools pygtftk

4. Download and install DoGFinder.

> git clone https://github.com/shalgilab/DoGFinder.git

> cd DoGFinder

> sudo python setup.py install

> Note: # Alternative command for non-root users

> python setup.py install --home=<dir>

5. Install the BioConductor R packages needed for the analysis.

> if (!requireNamespace("BiocManager", quietly = TRUE))

install.packages("BiocManager")

BiocManager::install(c("DESeq2", "ggplot2", "GGally", "ggpubr", "reshape2", "org.Mm.eg.db", "readxl", "data.table"))

## Key resources table

**Table:** 

REAGENT or RESOURCE	SOURCE	IDENTIFIER
**Chemicals, peptides, and recombinant proteins**		

FuGENE® HD reagent	Promega	Cat#E2311
Hygromycin B	Thermo Fisher Scientific	Cat#10687010
Geneticin	Gibco	Cat#10131035
RNA extraction reagent	Solarbio	Cat#P1014
TRIzol reagent	Thermo Fisher Scientific	Cat#15596026
cOmplete Protease Inhibitor EASYpacks EDTA-Free	Roche	Cat#4693159001
RNA extraction reagent	Solarbio	Cat#P1014

**Recombinant DNA**		

pX332 vector	Lab of Jiazhi Hu	N/A
Fluc-BoxB-Rluc vector	Lab of Yang Yu	N/A

**Deposited data**		

dRPB3 ChAR-seq data	Li et al.^1^	GEO: GSE225453
RPB3 ChAR-seq data	Li et al.^2^	GEO: GSE179962

**Experimental models: Cell lines**		

Mouse: V6.5 mES	Lab of Richard A. Young	N/A
Drosophila Schneider 2 (S2)	Lab of Jian Zhu	N/A

**Oligonucleotides**		

F-Rpb1-sgRNA: CACCG TGGTTAGGGTCAGACAACCT	Li et al.^1^	N/A
R-Rpb1-sgRNA: AAAC AGGTTGTCTGACCCTAACCA C	As above	N/A
F-Rpb1-genotyping: CTCCCCGACATACTCACCAACC	As above	N/A
R-Rpb1-genotyping: ACCAGCTCTTCGCCCTGTTCGCTCA	As above	N/A

**Software and algorithms**		

Bowtie2	Langmead and Salzberg^3^	http://bowtie-bio.sourceforge.net/bowtie2/index.shtml
Cutadapt v2.10	Martin^4^	https://cutadapt.readthedocs.io/en/stable/
FastQC	N/A	https://www.bioinformatics.babraham.ac.uk/projects/fastqc/
Sambamba v0.7.0	Tarasov et al.^5^	https://github.com/biod/sambamba
SAMtools v0.1.19	Li et al.^6^	http://www.htslib.org/
DeepTools v3.4.3	Ramirez et al.^7^	https://deeptools.readthedocs.io/
RseQC v4.0.0	Wang et al.^8^	http://rseqc.sourceforge.net/
WiggleTools v1.2	Zerbino et al.^9^	https://github.com/Ensembl/WiggleTools
Bedtools v2.29	Quinlan and Hall^10^	https://bedtools.readthedocs.io/en/latest/
DESeq2	Love et al.^11^	https://bioconductor.org/packages/release/bioc/html/DESeq2.html
Dogfinder	Wiesel et al.^12^	https://github.com/shalgilab/DoGFinder
Pygtftk	N/A	https://dputhier.github.io/pygtftk/index.html

## Materials and equipment

**Table:** NP-40 lysis buffer

Reagent	Final concentration	Amount
Tris-HCl (pH 7.5) (1 M)	10 mM	400 μL
NaCl (5 M)	150 mM	1.2 mL
NP-40	0.05%	20 μL
ddH2O	N/A	38.38 mL
**Total**	**N/A**	**40 mL**

**Table:** Sucrose cushion

Reagent	Final concentration	Amount
Sucrose	24%	9.2 g
NP-40 lysis buffer	N/A	40 mL
**Total**	**N/A**	**40 mL**

**Table:** Glycerol buffer

Reagent	Final concentration	Amount
Tris-HCl (pH 8.0) (1 M)	20 mM	800 μL
NaCl (5 M)	75 mM	600 μL
EDTA (500 mM)	0.5 mM	40 μL
DTT (1 M)	0.85 mM	34 μL
Glycerol	50%	20 mL
ddH2O	N/A	18.506 mL
**Total**	**N/A**	**40 mL**

**Table:** Nuclei lysis buffer

Reagent	Final concentration	Amount
HEPES (pH 7.6) (1 M)	10 mM	400 μL
NaCl (5 M)	300 mM	2.4 mL
EDTA (500 mM)	0.2 mM	16 μL
DTT (1 M)	1 mM	40 μL
MgCl2 (1 M)	7.5 mM	300 μL
Urea	1 M	2.4024 g
NP-40	1%	400 μL
ddH2O	N/A	36.444 mL
**Total**	**N/A**	**40 mL**

## Step-by-step method details

### Chromatin-associated RNA extraction with spike-in control

**Timing: ∼1–2 days**

Prepare chromatin-associated RNA according to protocol published previously.^13^

1. Pretreat mES cells with 1 μg/mL doxycycline for 12 h, treat RPB3 or dRPB3 degron mES cells with or without 500 μM indole-3-acetic acid (auxin/IAA).
2. Collect approximately 10ˆ7 cells and mix them with 10% Drosophila S2 cells as spike-in control.
3. Lyse the cells in ice-cold NP-40 lysis buffer for 5 min (10 mM Tris-HCl (pH 7.5), 150 mM NaCl, 0.05% NP-40, 1 × proteinase inhibitor cocktail).
4. Cell lysates were gently put on the top of the sucrose cushion (24% sucrose in NP-40 lysis buffer), centrifuged at 12,000 *g* for 10 min. Perform sucrose cushion precipitation to isolate nuclei. Discard the supernatants representing the cytoplasm and resuspend the pellets in 0.5 mL glycerol buffer.
5. Add 0.5 mL nuclei lysis buffer to lyse the nuclei and incubate on ice for 2 min.
6. Centrifuge the mixture and discard the supernatant representing the nucleoplasm fraction.
7. Discard the supernatant and washed twice with ice-cold PBS.
8. Extract the RNA from the chromatin pellet using TRIzol reagent according to the manufacturer’s manual. Troubleshooting 1.
9. Send the RNA samples to Novogene for ribosomal RNA depletion, strand-specific library construction, and sequencing.

***Alternatives:*** Cells from other species can also be used for normalization. We recommend using higher percentage of spike-in controls, such as 10%–20%.

***Note:*** In 1–9 steps, different samples should be processed at the same time by the same reagents and equipment to reduce batch effects.

### Quality control and sequence alignment

**Timing: ∼10 h**

**Figure 2:**
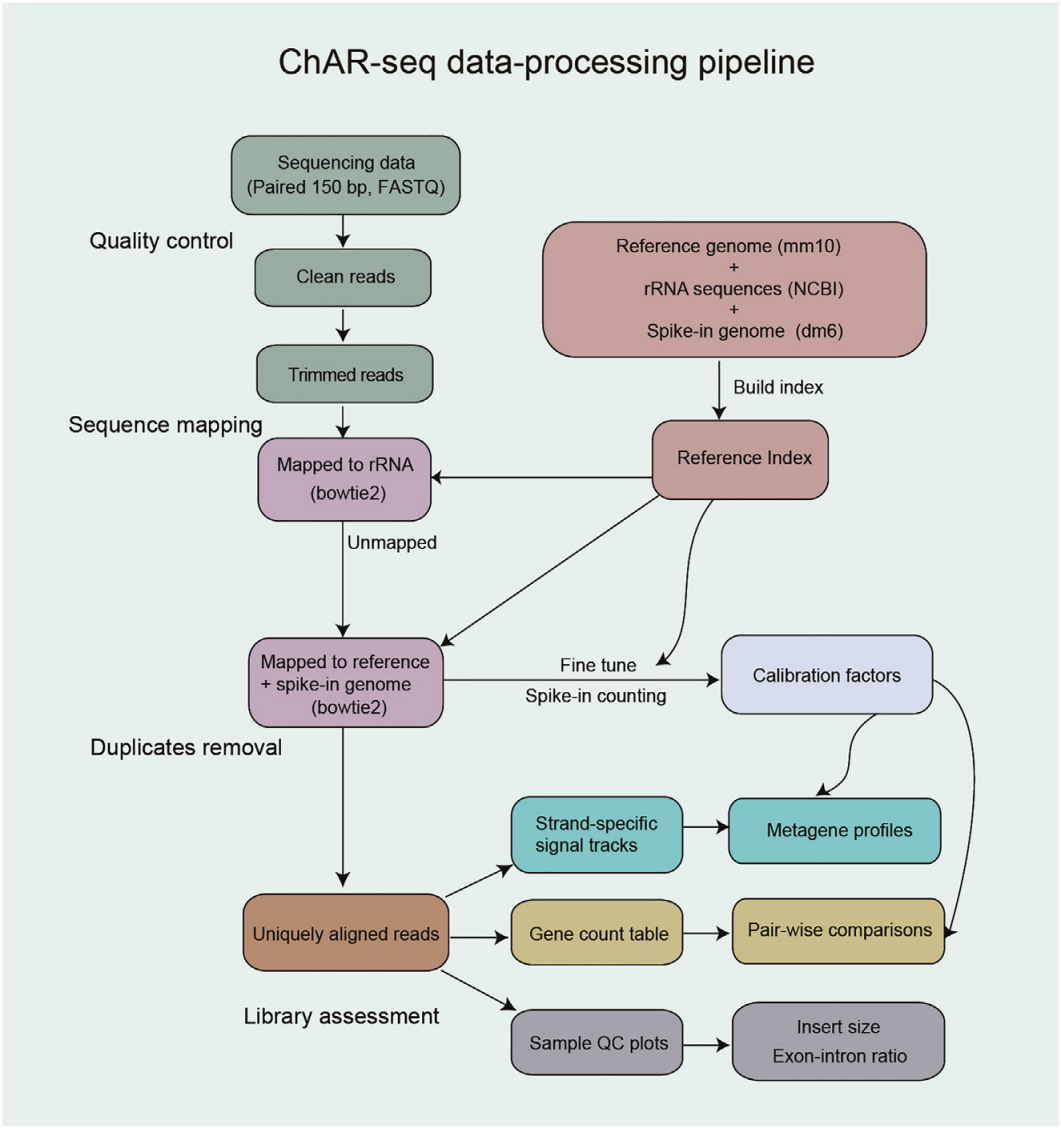
Overview of the ChAR-seq data-processing pipeline

The workflow for ChAR-seq data processing is shown in Figure 2.

The minimum computational requirement: this pipeline requires at least 10 GB of RAM.

10. Trim adapter and low-quality bases from the raw sequencing data using the cutadapt tool (v2.10) with customized settings. Low-quality bases with a Phred quality score of <15 will be trimmed and reads with a final length of <25 bp will be discarded.

#!/bin/bash

# Step 10: Trim adapters from paired-end FASTQ files.

# Note: Make sure cutadapt is installed before running this script.

# Set variables for the example of dRPB3_IAA_0h_rep1

outputdir="/path/to/output_directory"

forward_adapt="CCCCCCCCCAGATCGGAAGAGCACACGTCTGAACTCCAGTCAC"

reverse_adapt="AGATCGGAAGAGCGTCGTGTAGGGAAAGAGTGT"

min_length=25

prefix="dRPB3_IAA_0h_rep1"

fastq1="dRPB3_IAA_0h_rep1_R1.fq.gz"

fastq2="dRPB3_IAA_0h_rep1_R2.fq.gz"

# Create directory for cutadapt

mkdir -p "${outputdir}/cutadapt"

# Run cutadapt

cutadapt -a "${forward_adapt}" ∖

  -A "${reverse_adapt}" ∖

  -q 15,15 ∖

  --overlap 1 ∖

  -m "${min_length}" ∖

  -o "${outputdir}/cutadapt/${prefix}_1.fq" ∖

  -p "${outputdir}/cutadapt/${prefix}_2.fq" ∖

  "${fastq1}" "${fastq2}"

11. Check the quality of the trimmed reads using fastqc (v0.3.0).

# Step11: Run FastQC to check the quality of the trimmed reads

# Note: Step11 is part of a large script with the previous step.

# Variables are defined earlier in the same script.

# create directory for cutadapt output and quality control results

mkdir -p "${outputdir}/cutadapt/QC"

# run FastQC on the cutadapt output files

fastqc "${outputdir}/cutadapt/${Prefix}_1.fq" "${outputdir}/cutadapt/${Prefix}_2.fq" ∖

   -o "${outputdir}/cutadapt/QC"

12. Download rRNA sequences for *Mus musculus* from GenBank with accession number BK000964.3.
13. Build an index and map the trimmed reads to the rRNA sequence using the bowtie2 aligner (v2.3.5.1).

# Note: Steps 12 and 13 are parts of a large script with the previous step.

# Variables are defined earlier in the same script.

# create directory for genome sequence

mkdir -p "${outputdir}/genome/ "

# Define variables

bowtie2_index="${outputdir}/genome/mm10_rRNA_bowtie2_index"

rRNA_accession="BK000964.3"

# Step12: Download the rRNA sequence from GenBank

# Note: The 'efetch' command is used to download sequences from GenBank based on the accession number.

efetch -db nucleotide -id "${rRNA_accession}" -format fasta > "${outputdir}/genome/mm10_rRNA.fasta"

# Step13: Build the index and map trimmed reads to the rRNA sequence and keep unmapped reads

bowtie2-build "${outputdir}/genome/mm10_rRNA.fasta" "${bowtie2_index}"

bowtie2 -x "${bowtie2_index}" -U "${trimmed_reads}" ∖

  --un "${outputdir}/unmapped_reads.fastq" ∖

  -S "${outputdir}/mapped_reads.sam"

14. Map the unmapped reads from the output of bowtie2 to the concatenated reference genome comprised of mouse and Drosophila chromosomes (mm10 + dm6) using bowtie2 with options (-very-sensitive-local).
  a. Download the mouse (GRCm38/mm10) and Drosophilareference genome sequences (dm6) from the UCSC genome browser and saved as “mm10.fa.gz” and “dm6.fa.gz”, respectively.
  b. Uncompress “mm10.fa.gz” and “dm6.fa.gz”.
  c. Because both mouse and Drosophila genomes have chromosome X (named “chrX”) and chromosome Y (named “chrY”), we need to modify the *Drosophila* chromosome IDs to make them different from that of the mouse. In this case, we add “dm6_” to each chromosome ID of the *Drosophila.* For example, we change *Drosophila*’s “chrX” into “dm6_chrX”, “chr2L” into “dm6_chr2L”, and so forth.
  d. Concatenate the mouse (mm10) and Drosophilareference genome sequences (mm10) into a single FASTA file – the composite reference genome named “mm10_dm6.fa”.
  e. Index the “mm10_dm6.fa” using the “bowtie2-build” command.
  f. Map reads to the composite reference genome using Bowtie2. Using Samtools, convert the output alignments into BAM format, sort the BAM files by position, and then index them.
  g. Repeat step f for all biological replicates of ChAR-seq data in RPB3- and dRPB3-degron cells before and after IAA treatment.

# Note: Steps14(a)-(e) are parts of a large script with the previous step.

# Variables are defined earlier in the same script.

# Define variables

unmapped_reads="${outputdir}/unmapped_reads.fastq"

mouse_reference="mm10.fa"

drosophila_reference="dm6.fa"

composite_reference="mm10_dm6.fa"

bowtie2_index="${outputdir}/mm10_dm6_bowtie2_index"

# Step14a: Download the mouse and Drosophila reference genome sequences from the UCSC genome browser

wget http://hgdownload.soe.ucsc.edu/goldenPath/mm10/bigZips/${mouse_reference}.gz -P "${outputdir}"

wget http://hgdownload.soe.ucsc.edu/goldenPath/dm6/bigZips/${drosophila_reference}.gz -P "${outputdir}"

# Step14b: Uncompress the reference genome sequences

gunzip "${outputdir}/${mouse_reference}.gz"

gunzip "${outputdir}/${drosophila_reference}.gz"

# Step14c: Modify the Drosophila chromosome IDs

sed -i 's/ˆ>chr∖([0-9A-Za-z]∖)/>dm6_chr∖1/' "${outputdir}/${drosophila_reference}"

# Step14d: Concatenate the mouse and Drosophila reference genome sequences into a single FASTA file

cat "${outputdir}/${mouse_reference}" "${outputdir}/${drosophila_reference}" > "${outputdir}/${composite_reference}"

# Step 5e: Index the composite reference genome using the bowtie2-build command

bowtie2-build "${outputdir}/${composite_reference}" "${outputdir}/${bowtie2_index}"

15. Remove unmapped or unpaired reads and sort and generate indexes of filtered bam files using sambamba (v0.7.0) and SAMtools (v0.1.19).
16. Classify reads by splitting the "composite" BAM files into mouse and Drosophila BAM files.

# Note: Steps15 and16 are parts of a large script with the previous step.

# Variables are defined earlier in the same script.

# create directory for mapping results

mkdir -p "${outputdir}/mapping"

# map reads to reference and spike-in genome using Bowtie2

bowtie2 -q -x "${index}" --very-sensitive-local --reorder -1 "${outputdir}/cutadapt/${Prefix}_1.fq" -2 "${outputdir}/cutadapt/${Prefix}_2.fq" -p 15 -S "${outputdir}/mapping/${Prefix}.sam" 2> "${outputdir}/mapping/${Prefix}_mapping.stat"

# Step15: mark duplicates using sambamba

# Note: Sambamba is used to mark duplicates in the mapped reads.

# The '-r' option removes secondary and supplementary alignments, '-t' specifies the number of threads.

sambamba markdup -r -t 10 "${outputdir}/mapping/${Prefix}.bam" "${outputdir}/mapping/${Prefix}_rmdup.bam"

# Step15: sort the SAM file and convert it to BAM format

# Note: The SAM file is sorted and converted to BAM format using samtools mentioned in Step15.

# The '-@' option specifies the number of threads, '-o' specifies the output BAM.

samtools sort -@ 10 -o "${outputdir}/mapping/${Prefix}.bam" "${outputdir}/mapping/${Prefix}.sam"

# extract unique and concordant pairs from the BAM file

echo "Extracting unique and concordant pairs..."

samtools view -@ 5 -hF 4 "${outputdir}/mapping/${Prefix}_rmdup.bam" | grep -v "XS:" | samtools view -@ 5 -bS -o "${outputdir}/mapping/${Prefix}_unique.bam"

samtools view -H "${outputdir}/mapping/${Prefix}_rmdup.bam" > "${outputdir}/mapping/header.txt"

samtools view -@ 5 -hF 4 "${outputdir}/mapping/${Prefix}_unique.bam" | grep "YT:Z:CP" | cat "${outputdir}/mapping/header.txt" - | samtools view -@ 5 -bS -o "${outputdir}/mapping/${Prefix}_unique_concordant.bam"

samtools index "${outputdir}/mapping/${Prefix}_unique_concordant.bam"

# Step16: split the "composite" BAM files into mouse and Drosophila BAM files

echo "Filtering out reads that map to non-target regions..."

samtools view -h "${outputdir}/mapping/${Prefix}_unique_concordant.bam" $(echo dm6_chr{2L,2R,3L,3R,4,X,Y,M}) -o "${outputdir}/mapping/${Prefix}_unique_concordant_dm6.bam"

samtools view -h "${outputdir}/mapping/${Prefix}_unique_concordant.bam" $(echo chr{{1..19},X,Y,M}) -o "${outputdir}/mapping/${Prefix}_unique_concordant_mm10.bam"

17. Count reads originating from Drosophila S2 spike-in cells and calculate the calibration factors (alpha=1e6/dm6_count) for reads that mapped to the mouse genome.

>scale_factor=$(echo 1000000/${fly_num} | bc -l)

18. Assign plus and minus strands to uniquely mapped reads using SAMtools.

# Step18: Use SAMtools to assign plus and minus strands for uniquely mapped reads

# Note: Step18 is part of a large script with the previous step.

# Variables are defined earlier in the same script.

# Create output directory

mkdir -p "$output_dir/mapping/split_strand"

# Split bam file by strand

samtools view -@ 10 -hf 80 -F 32 "$input_bam" -o "$output_dir/mapping/split_strand/${prefix}_r1_rev.bam"

samtools view -@ 10 -hf 96 -F 16 "$input_bam" -o "$output_dir/mapping/split_strand/${prefix}_r1_forw.bam"

samtools view -@ 10 -hf 144 -F 32 "$input_bam" -o "$output_dir/mapping/split_strand/${prefix}_r2_rev.bam"

samtools view -@ 10 -hf 160 -F 16 "$input_bam" -o "$output_dir/mapping/split_strand/${prefix}_r2_forw.bam"

# Merge and sort strand-specific bam files

samtools merge -f -@ 5 "$output_dir/mapping/split_strand/${prefix}_plus.bam" "$output_dir/mapping/split_strand/${prefix}_r1_rev.bam" "$output_dir/mapping/split_strand/${prefix}_r2_forw.bam"

samtools sort -@ 5 -o "$output_dir/mapping/split_strand/${prefix}_plus_sorted.bam" "$output_dir/mapping/split_strand/${prefix}_plus.bam"

samtools index "$output_dir/mapping/split_strand/${prefix}_plus_sorted.bam"

rm "$output_dir/mapping/split_strand/${prefix}_plus.bam"

samtools merge -f -@ 5 "$output_dir/mapping/split_strand/${prefix}_minus.bam" "$output_dir/mapping/split_strand/${prefix}_r1_forw.bam" "$output_dir/mapping/split_strand/${prefix}_r2_rev.bam"

samtools sort -@ 5 -o "$output_dir/mapping/split_strand/${prefix}_minus_sorted.bam" "$output_dir/mapping/split_strand/${prefix}_minus.bam"

samtools index "$output_dir/mapping/split_strand/${prefix}_minus_sorted.bam"

rm "$output_dir/mapping/split_strand/${prefix}_minus.bam"

19. Convert final BAM files to bigWig tracks in consecutive 10 bp throughout the genome, separated by strand, and normalize to spike-in controls using bamCoverage from deeptools.

# Step19: Generate bigWig files for plus and minus strand reads

# Note: Step19 is part of a large script with the previous step.

# Variables are defined earlier in the same script.

bamCoverage --bam ${outputdir}/mapping/split_strand/${Prefix}_plus_sorted.bam ∖

-o ${outputdir}/bigwig/${Prefix}_plus_spikein.bw ∖

--binSize 10 ∖

--normalizeUsing None ∖

--scaleFactor ${scale_factor} ∖

--effectiveGenomeSize 2652783500 ∖

-p 5

bamCoverage --bam ${outputdir}/mapping/split_strand/${Prefix}_minus_sorted.bam ∖

-o ${outputdir}/bigwig/${Prefix}_minus_spikein.bw ∖

--binSize 10 ∖

--normalizeUsing None ∖

--scaleFactor ${scale_factor} ∖

--effectiveGenomeSize 2652783500 ∖

-p 5

20. Merge the bigWig score tracks from two biological replicates to an averaged signal for visualization using the mean operator from the WiggleTools (v1.2) package.

# Note: Step20 is standalone and function independently.

# Variables are defined within this script.

#Define the list of samples, stages and strands as arrays

samples=("RPB3_IAA" "dRPB3_IAA")

stages=("0h" "1h" "3h")

strands=("plus" "minus")

genome_file="mm10_dm6.genome.txt"

# Use arrays to loop through the values

for sample in "${samples[@]}"

do

 for stage in "${stages[@]}"

 do

  for strand in "${strands[@]}"

  do

   # Use variables to make the command more readable input_file_1="${output_dir}/bigwig/${sample}_${stage}_1_${strand}_spikein.bw"

input_file_2="${output_dir}/bigwig/${sample}_${stage}_2_${strand}_spikein.bw"

   output_file="${sample}_${stage}_${strand}_spikein.bw"

   # Note: The 'wiggletools mean' calculates the mean of the bigWig files, and the output is redirected to 'wigToBigWig' along with the genome file and output file path. wigToBigWig <(wiggletools mean "$input_file_1" "$input_file_2") "$genome_file" "${output_dir}/bigwig/${output_file}"

   done

  done

done

### Evaluate ChAR-seq library with summary statistics

**Timing: ∼4 h**

**Figure 3:**
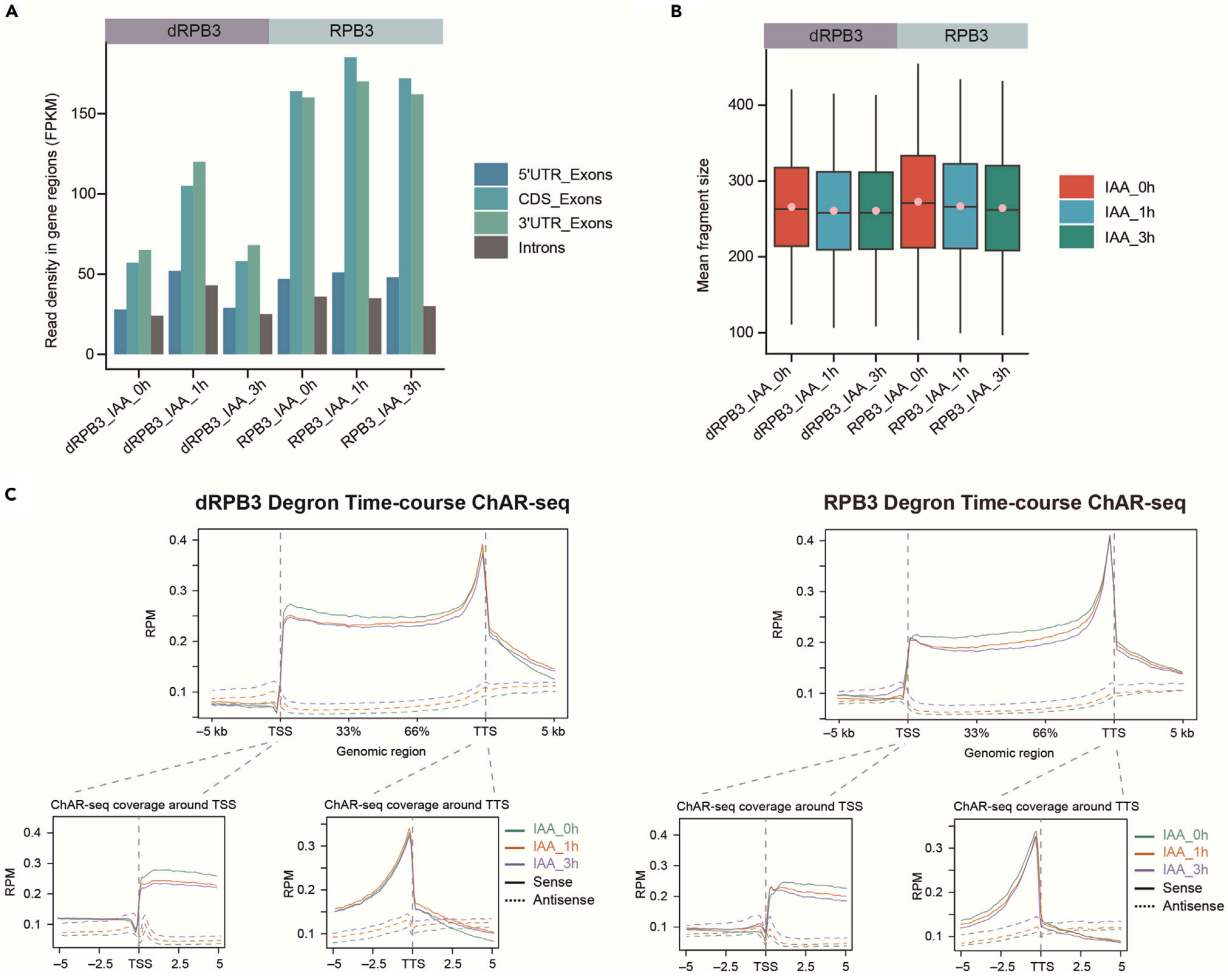
Assessment of ChAR-seq library (A) Distribution of uniquely mapped read pairs over gene features (5′UTR, CDS, 3′UTR, exon, and intron) across six merged ChAR-seq samples. (B) Distribution of fragment sizes of different samples, inferred from the separation of read pairs using RSeQC. (C) Metagene profiles of normalized ChAR-seq reads in gene bodies at each time point after IAA treatment. The vertical dashed lines indicate the magnified portions of TSS (transcription start site, left) and TTS (transcription termination site, right) regions. ChAR-seq densities in the sense and antisense directions are indicated by solid and dashed lines, respectively. Figure adapted from Li et al.^2^

Evaluate the ChAR-seq sequencing library with mapped reads distribution, RNA fragment size and metagene profile (Figure 3).

21. Inspect the distribution of mapped reads and read density over gene features using the read_distribution.py module from RSeQC.
22. Prioritize gene features as follows: CDS exons > UTR exons > Introns.
23. Calculate the inner distance between read pairs to estimate the RNA fragment size using the inner_distance.py module.
24. Record the calculated mean, median, and standard deviation values for QC plots.

# Note: Steps21 and23 are standalone and function independently.

# Variables are defined within this script.

# Define the list of samples and stages as arrays

samples=("RPB3_IAA" "dRPB3_IAA")

stages=("0h" "1h" "3h")

# Loop through each sample and stage

for sample in "${samples[@]}"

do

 for stage in "${stages[@]}"

 do

  # Define the input and output filenames

  input_bam="${sample}_${stage}.bam"

  output_inner="${sample}_${stage}.inner_distance"

# Step21: Inspect the distribution of mapped reads and read density over gene features

# Note: The 'read_distribution.py' module from RSeQC is used to analyze the distribution of mapped reads over gene features.

  read_distribution.py -i "$input_bam" -r mm10_RefSeq.bed >> read_distribution.txt

# Step23: Calculate inner distance between read pairs to estimate RNA fragment size

# Note: The 'inner_distance.py' module from RSeQC is used to calculate the inner distance between read pairs.

  inner_distance.py -i "$input_bam" -o "$output_inner" -q 0 -r mm10_RefSeq.bed

 done

done

25. Report the number of aligned reads and the alignment percentage as the alignment statistics (Table 1)

**Table 1:** Chromatin-associated RNA sequencing mapping statistics

Samples (paired-end)	Total read pairs	Trimmed reads	Mapping reads	rm duplicate	Unique concordant pairs	mm10 concordant pairs	dm6 concordant pairs
dRPB3_IAA_0h_rep1	37,108,432	99.99%	84.42%	55.46%	45.71%	44.77%	0.94%
dRPB3_IAA_0h_rep2	41,355,443	99.99%	82.98%	53.64%	43.89%	42.96%	0.93%
dRPB3_IAA_1h_rep1	34,980,539	99.99%	83.56%	54.85%	44.89%	43.95%	0.94%
dRPB3_IAA_1h_rep2	39,292,149	99.99%	82.96%	52.92%	43.29%	42.40%	0.89%
dRPB3_IAA_3h_rep1	50,504,311	99.99%	83.02%	49.78%	40.69%	39.85%	0.84%
dRPB3_IAA_3h_rep2	39,688,559	99.99%	82.97%	53.35%	43.81%	42.93%	0.87%
RPB3_IAA_0h_rep1	35,296,310	100.00%	96.74%	64.77%	40.03%	39.45%	0.58%
RPB3_IAA_0h_rep2	37,342,429	100.00%	96.65%	63.84%	39.55%	38.87%	0.68%
RPB3_IAA_1h_rep1	34,118,478	100.00%	95.81%	61.26%	38.47%	37.80%	0.67%
RPB3_IAA_1h_rep2	47,730,115	100.00%	95.78%	57.49%	34.89%	34.13%	0.77%
RPB3_IAA_3h_rep1	35,739,215	100.00%	96.35%	63.43%	38.78%	37.97%	0.81%
RPB3_IAA_3h_rep2	32,886,424	100.00%	97.07%	67.24%	40.42%	39.36%	1.05%

# Step25: Record alignment statistics

# Note: Step25 is part of a large script along with the step19.

# Variables are defined earlier in the same script.

# Count the number of reads using the appropriate commands

cutadapt=$(grep "ˆ@" ${outputdir}/cutadapt/${Prefix}_1.fq | wc -l)

mapping=$(samtools view -F 4 ${outputdir}/mapping/${Prefix}.bam | wc -l)

rmdup=$(samtools view -F 4 ${outputdir}/mapping/${Prefix}_rmdup.bam | wc -l)

unique=$(samtools view -c ${outputdir}/mapping/${Prefix}_unique.bam)

concordant=$(samtools view -c ${outputdir}/mapping/${Prefix}_unique_concordant.bam)

mm10_concord=$(samtools view -c ${outputdir}/mapping/${Prefix}_unique_concordant_mm10.bam)

dm6_concord=$(samtools view -c ${outputdir}/mapping/${Prefix}_unique_concordant_dm6.bam)

# Write the statistics to the stastic file

echo -e "sample_name,total_reads,cutadapt,mapping reads,rm duplicate,unique mapping,concordant pairs,mm10 concordant pairs,dm6 concordant pairs" >> ${outputdir}/${Prefix}_stastic.csv

echo -e "${Prefix},${total_reads},${cutadapt},${mapping},${rmdup},${unique},${concordant},${mm10_concord},${dm6_concord}" >> ${outputdir}/${Prefix}_stastic.csv

26. Generate strand-specific metagene, TSS and TTS profiles from a BAM file using "ngs.plot".
  a. Set working directories, sample information and their corresponding BAM file location.
    #!/bin/bash
    # Note: Step26a are standalone and function independently.
    # Variables are defined within this script.
    # Set working directory, temporary directory, and metaprofiles directory
    WORKDIR="/path/to/my/working_directory/"
    # Note: $WORKDIR is where the BAM files to be plotted are stored.
    TMPDIR="${WORKDIR}tmp/"
    PROFDIR="${WORKDIR}metaprofiles/"
    # Create temporary and metaprofiles directories
    mkdir -p "$TMPDIR"
    mkdir -p "$PROFDIR"
    # Define the sample name and input BAM file
    SAMPLE="dRPB3_0h"
    BAM="${WORKDIR}dRPB3_0h.bam"
    # Define the output BAM file with mate pairs
    MATE1="${WORKDIR}dRPB3_0h.mate1.bam"
  b. Restrict to the first mate reads. If the BAM file contains paired reads, create a new file containing only the first mate reads.
  c. Reformat the chromosome names in the BAM file to match those in the ngs.plot database.
    # Step26c: Reformat the chromosome names
    # Note: Step26c is part of a large script along with the step26a.
    # Variables are defined earlier in the same script.
    # Define output file names and the number of threads
    MATE1REHEADER="${WORKDIR}/dRPB3_0h.mate1.reheader.bam"
    THREADS=10
    # Convert BAM to SAM, filter by flag 64, and convert back
    samtools view --threads $THREADS -h -b -f 64 $BAM -o $MATE1
    # Index the BAM file
    samtools index $MATE1
    # Modify the header of the BAM file to replace chromosome numbers with "chr" prefix
    samtools view --threads $THREADS -H ${MATE1} | sed -e 's/SN:∖([0-9XY]∗∖)/SN:chr∖1/' -e 's/SN:MT/SN:chrM/' | samtools reheader - ${MATE1} > ${MATE1REHEADER}
    # Index the reheadered BAM file
    samtools index ${MATE1REHEADER}
  d. Run ngs.plot to generate sense and anti-sense profiles for specific regions of interest using the correct BAM file. Note that if the libraries were produced such that mate2 represents the forward strand, the sense and anti-sense profiles would be reversed.
    # Note: Step26d is part of a large script along with the step26a and26c.
    # Variables are defined earlier in the same script.
    ## Genebody
    REGION="genebody"
    for STRAND in both same opposite
    do
     OUTPUT="${PROFDIR}${SAMPLE}.${REGION}.${STRAND}"
    ngs.plot.r -G mm10 -R $REGION -C ${MATE1REHEADER} -O $OUTPUT -P 10 -SS $STRAND -SE 1 -L 5000 -F charseq -GO total -RB 0.05 -AL bin -CS 500 -FL 150 -D ensembl
    done
    ## TSS
    REGION="tss"
    for STRAND in both same opposite
    do
     OUTPUT="${PROFDIR}${SAMPLE}.${REGION}.${STRAND}"
    ngs.plot.r -G mm10 -R $REGION -C ${MATE1REHEADER} -O $OUTPUT -P 10 -SS $STRAND -SE 1 -L 5000 -F charseq -GO total -RB 0.05 -AL bin -CS 500 -FL 150 -D ensembl
    done
    ## TTS
    REGION="tts"
    for STRAND in both same opposite
    do
     OUTPUT="${PROFDIR}${SAMPLE}.${REGION}.${STRAND}"
    ngs.plot.r -G mm10 -R $REGION -C ${MATE1REHEADER} -O $OUTPUT -P 10 -SS $STRAND -SE 1 -L 5000 -F charseq -GO total -RB 0.05 -AL bin -CS 500 -FL 150 -D ensembl
    done
  e. Use R to combine the sense and anti-sense profiles on a single set of axes by utilizing the ngs.plot output.
  f. Scale the profile by employing the calculated calibration factors in **step** 17**.**
  g. Edit the resulting ".cnt" file to multiply the read count by the calibration factor, and re-run ngs.plot using the same parameters as before with the modified one.
    # This R code refers to plots inFigure 3
    # Load required packages
    library(ggplot2)
    library(reshape2)
    library(ggpubr)
    library(readxl)
    # Read the Excel file and remove missing values forFigure 3A
    df <- na.omit(read_excel("protocol_stats.xlsx", sheet = 1, trim_ws = FALSE, range = cell_cols(c("A", "E"))))
    # Reshape the data using the melt function from the reshape2 package
    df_long <- reshape2::melt(df, id = "Samples", value.name = "count")
    # Round the count values to the nearest integer
    df_long$count <- round(df_long$count, 0)
    # Create a grouped bar plot using ggbarplot from ggpubr
    p1 <- ggbarplot(data = df_long, x = "Samples", y = "count",
        fill = "variable", palette = c('#3494BAFF', '#58B6C0FF', '#75BDA7FF', '#666666FF'),
        position = position_dodge(0.7),
        # Set background and line colors
        background.color = "white", color = NA,
        xlab = "", ylab = "FPKM",
        remove = "all",
        axis.line = element_line(color = "black")
    ) + ggpubr::theme_pubr()
    # Save the plot as a PDF file
    ggsave(p1, filename = "Figure 3A_gene_features.pdf", width = 6, height = 4)
    # Read the Excel file and remove missing values forFigure 3B
    df <- na.omit(read_excel("protocol_stats.xlsx", sheet = 2, trim_ws = FALSE, range = cell_cols(c("F", "J"))))
    # Create the second plot
    p2 <- ggplot(df, aes(x = Samples, y = Mean, fill = Group)) +
     geom_boxplot(aes(lower = Mean - SD, upper = Mean + SD, middle = Median, ymin = Mean - 3 ∗ SD, ymax = Mean + 3 ∗ SD),
         stat = "identity", width = 0.8) +
     stat_summary(fun.y = mean, geom = "point", shape = 20, size = 3, color = "pink", fill = "pink") +
     scale_fill_manual(values = c("#E64B35FF","#4DBBD5FF","#00A087FF"))+
     ggpubr::theme_pubr()
    # Save the box plot as a PDF file
    ggsave(p2, filename = "Figure 3B_insert size.pdf", width = 5, height = 4)
    # The following R code refers to metagene plot for dRPB3 inFigure 3C
    # Note: similar code can be employed for RPB3.
    # Define input files forFigure 3C
    sense_profile <- "dRPB3_sense_avgprof.txt"
    antisense_profile <- "dRPB3_antisense_avgprof.txt"
    # Read sense and anti-sense profiles from ngs.plot output
    sense <- read.table(sense_profile, header = TRUE)
    antisense <- read.table(antisense_profile, header = TRUE)
    # Plot the combined profiles
    pdf("Figure 3C_metagene_dRPB3.pdf", 8,8)
    plot(sense$position, sense$IAA_0h, type = "l", col = "#1B9E77", lwd = 2,
     xlab = "Position", ylab = "Value", main = "Combined Profiles")
    lines(sense$position, sense$IAA_1h, type = "l", col = "#D95F02", lwd = 2)
    lines(sense$position, sense$IAA_3h, type = "l", col = "#7570B3", lwd = 2)
    lines(antisense$position, antisense$IAA_0h, type = "l", col = "#1B9E77", lwd = 2)
    lines(antisense$position, antisense$IAA_1h, type = "l", col = "#D95F02", lwd = 2)
    lines(antisense$position, antisense$IAA_3h, type = "l", col = "#7570B3", lwd = 2)
    legend("topright", legend = c("Sense IAA_0h", "Sense IAA_1h", "Sense IAA_3h",
            "Anti-sense IAA_0h", "Anti-sense IAA_1h", "Anti-sense IAA_3h"),
       col = c("#1B9E77", "#D95F02", "#7570B3", "#1B9E77", "#D95F02", "#7570B3"), lty = 1, lwd = 2)
    dev.off()

### Construct gene loci annotation and down-sample the resulting bam files

**Timing: ∼6 h**

27. Download the mouse gene annotation GTF file from “Comprehensive gene annotation” in the “GTF/GFF3 files” table named “gencode.vM23.annotation.gtf.gz”, which is the main annotation file for most users.

> wget -c https://ftp.ebi.ac.uk/pub/databases/gencode/Gencode_mouse/release_M23/gencode.vM23.annotation.gtf.gz

28. Unzip gene annotation files.

> gunzip gencode.vM23.annotation.gtf.gz

29. Check the strandedness of comma-separated GTF files, and use it as input to generate a gene loci annotation file.

> Get_loci_annotation -out ./gencode_mm10_vM23 -gtf gencode.vM23.annotation.gtf

***Note:*** The output file is named loci_annotation.bed, to be used as input for Get_DoGs

30. Preprocess, sort and index the input bam files to keep their order constant.

#!/bin/bash

# Note: Step 30 are standalone and function independently.

# Variables are defined within this script.

# Define the list of samples, stages, and repeats as arrays

samples=("RPB3_IAA" "dRPB3_IAA")

stages=("0h" "1h" "3h")

reps=("rep1" "rep2")

# Loop over samples, stages, and replicates to perform samtools operations

for sample in "${samples[@]}"; do

 
for stage in "${stages[@]}"; do

 
 
for rep in "${reps[@]}"; do

 
 
 
# Perform samtools operations on the current sample, stage, and repeat

 
 
 
samtools view -@ 10 -hF 4 ${sample}_${stage}_${rep}.bam | grep "YT:Z:CP" | cat common_header.txt - | samtools view -@ 10 -bS -o ${sample}_${stage}_${rep}_reheader.bam

 
 
 
samtools sort ${sample}_${stage}_${rep}_reheader.bam -o ${sample}_${stage}_${rep}.bam

 
 
 
samtools index ${sample}_${stage}_${rep}.bam

 
 
 
done

 
done

done

31. Downsample the ChAR-seq bam files to match the read depth of the identified bam file with the lowest read depth.

> Pre_Process -Q 10 -bam dRPB3_IAA_0h_rep1.bam, dRPB3_IAA_0h_rep2.bam, dRPB3_IAA_1h_rep1.bam, dRPB3_IAA_1h_rep2.bam, dRPB3_IAA_3h_rep1.bam, dRPB3_IAA_3h_rep3.bam, RPB3_IAA_0h_rep1.bam, RPB3_IAA_0h_rep2.bam, RPB3_IAA_1h_rep1.bam, RPB3_IAA_1h_rep2.bam, RPB3_IAA_3h_rep1.bam, RPB3_IAA_3h_rep3.bam -ref ./gencode_mm10_vM23/loci_annotation.bed

***Note:*** Input sorted bam file of each sample should be separated by comma, troubleshooting 3.

***Note:*** This step may take a long time. Once finished, the output raw and downsampled files will be located in the same folder as the original bam files.

### Detect readthrough gene candidates

**Timing: ∼2 h**

32. Use the downsampled bam files and a loci annotation bed file as input for the DoGFinder tool to identify readthrough gene candidates.
  a. Remove all genic reads from the bam file.
  b. Limit gene boundaries by the location of the nearest 3‘ neighboring gene in the genome, and discard genes with 3’ nearest neighbor closer than 4 kb.
  c. Run the Get_DoGs function with the following parameters to identify readthrough gene candidates based on a minimum length of 4 kb and a minimum coverage of 60% over the entire downstream length: -S -minDoGLen 4000 -mode F -minDoGCov 0.6.
  d. Elongate the identified readthrough gene candidates to find their putative endpoint.

#!/bin/bash

# Note: Step 32 are standalone and function independently.

# Variables are defined within this script.

# Usage: Get_DoGs -out /outdir -bam my_sorted_bam.sorted_DS.bam -a loci_annotation.bed

# This script executes the Get_DoGs command for multiple samples, stages, and replicates.

# Define variables

samples=("dRPB3_IAA" "RPB3_IAA")

stages=("0h" "1h" "3h")

reps=("rep1" "rep2")

# Loop over samples, stages, and replicates to call the Get_DoGs command

for sample in "${samples[@]}"; do

 
for stage in "${stages[@]}"; do

 
 
for rep in "${reps[@]}"; do

 
 
 
Get_DoGs -out ./Get_DoGs ∖

 
 
 
 
 
 
-bam

"${sample}_${stage}_${rep}.sorted_PE.sorted_DS.bam" ∖

 
 
 
 
 
 
-suff "${sample}_${stage}_${rep}" ∖

 
 
 
 
 
 
-s -minDoGLen 4000 -minDoGCov 0.6 -w 200 -mode F ∖

 
 
 
 
 
 
-a ./gencode_mm10_vM23/loci_annotation.bed ∖

 
 
 
 
 
 
-max 100000 done

 
 
done

done

33. Intersect readthrough gene candidates from different replicates.
  a. Gather all the annotation bed files of readthrough gene candidates from different replicates that need to be intersected.
  b. Use the " Common_DoGs_annotation" function from DoGFinder to get the list of readthrough candidate gene sets common to all biological replicates.
  c. Execute the "sortBed" function from bedtools to sort the intersected bed file by chromosome and coordinate.
  d. Choose the most downstream coordinate as the end coordinate for all intersected readthrough gene candidates. Use the "awk" command to extract the most downstream coordinate for each gene name and save it in a new bed file.

#!/bin/bash

# Note: Step 33 are standalone and function independently.

# Variables are defined within this script.

# Usage: Common_DoGs_annotation -comm Dog_annotation_replicate1.bed,Dog_annotation_replicate2.bed -out /outdir

# This script executes the Common_DoGs_annotation command for multiple samples and stages.

# Define variables

samples=("dRPB3_IAA" "RPB3_IAA")

stages=("0h" "1h" "3h")

# Loop over samples and stages to run the Common_DoGs_annotation command

for sample in "${samples[@]}"; do

 
for stage in "${stages[@]}"; do

 
 
Common_DoGs_annotation -comm "Dog_annotation_${sample}_${stage}_rep1.bed, Dog_annotation_${sample}_${stage}_rep2.bed" ∖

 
 
 
 
 
 
 
 
./Final_Common_Dog_annotation/

 
 
sortBed -

i ./Final_Common_Dog_annotation/union_dog_annotation.bed ∖

> ./Final_Common_Dog_annotation/sorted_union_dog_annotation.bed

cat ./Final_Common_Dog_annotation/sorted_union_dog_annotation.bed ∖

 
 
| awk -F '∖t' -v OFS='∖t' '{if($4!=id) {print chr, start, end, id, score, strand; id=$4; chr=$1; start=$2; end=$3; score=$5; strand=$6;} else {if($3>end) end=$3;}} END {print chr, start, end, id, score, strand;}' ∖

 
 
> ./Final_Common_Dog_annotation/Final_CommonDog_annotation_${sample}_${stage}_rep1.bed

 
done

done

***Note:*** Assuming that the filenames for the replicate 1 and replicate 2 outputs follow the same pattern as the inputs,

34. Merge DoG annotations from different treatments or experiments.
  a. Collect all DoG annotation bed files to be merged for different treatments.
  b. Use the " Union_DoGs_annotation" function from DoGFinder to create a single DoG annotation bed file.
  c. For any DoG that appears in more than one input file, unify them according to their maximal length.
  d. Execute the "sortBed" function from bedtools to sort the merged bed file by chromosome and coordinate.
  e. Format the output bed file as a tab-separated gene annotation bed file to be used as input for Get_DoGs

#!/bin/bash

# Note: Step 34 is standalone and function independently.

# Variables are defined within this script.

# Usage: Union_DoGs_annotation -dog Final_Dog_annotation1.bed,Final_Dog_annotation2.bed -out /outdir

# This script executes the Union_DoGs_annotation command for multiple samples.

# Define variables

samples=("dRPB3_IAA" "RPB3_IAA")

# Create directories and run the Union_DoGs_annotation command

for sample in "${samples[@]}"; do

 
mkdir -p "./Final_UnionDog_annotation_${sample}"

 
Union_DoGs_annotation -dog "./Final_Common_Dog_annotation/Final_CommonDog_annotation_${sample}_0h.bed,./Final_Common_Dog_annotation/Final_CommonDog_annotation_${sample}_1h.bed,./Final_Common_Dog_annotation/Final_CommonDog_annotation_${sample}_3h.bed" ∖

-out "./Final_UnionDog_annotation_${sample}/"

cat "./Final_UnionDog_annotation_${sample}/union_dog_annotation.bed" | awk -F '∖t' -v OFS='∖t' '{print $1,$2,$3,$6,$5,$4 }' > "./Final_UnionDog_annotation/Final_UnionDog_annotation_${sample}.bed"

done

35. Calculate the expression levels of readthrough gene candidates across experiments.

**Figure 4:**
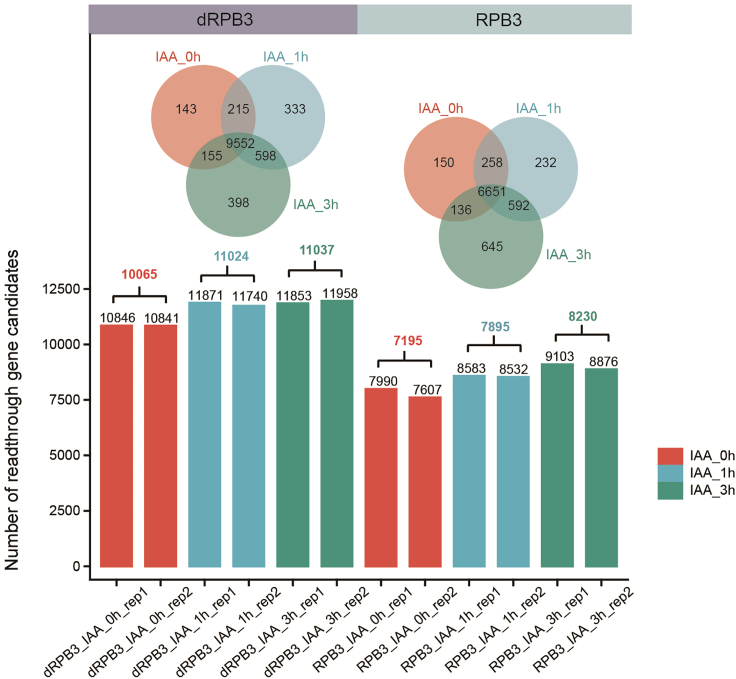
Comparison of readthrough gene candidates in dRPB3 and RPB3 degron cells treated with IAA Top: Venn diagram illustrating the union overlap between genes that produce readthrough genes at different time points (0 h, 1 h, and 3 h) after IAA treatment in dRPB3 (left) or RPB3 (right) degron cells. Bottom: Bar plot showing the number of readthrough gene candidates discovered by DoGFinder in each sample. The number in parentheses represents the common genes found in two replicates across six conditions.

#!/bin/bash

# Note: Step 35 is standalone and function independently.

# Variables are defined within this script.

# Usage: Get_DoGs_rpkm -out /outdir -bam my_sorted_bam_DS.bam -s -dog Final_Dog_annotation.bed

# This script calculates RPKM values for DoGs using the Get_DoGs_rpkm command.

# Define variables

samples=("dRPB3_IAA" "RPB3_IAA")

stages=("0h" "1h" "3h")

reps=("rep1" "rep2")

output_dir="./Get_DoGs_rpkm"

union_dog_file="./Final_UnionDog_annotation/Final_UnionDog_annotation_"

# Loop over samples, stages, and replicates to run the command

for sample in "${samples[@]}"; do

 
for stage in "${stages[@]}"; do

 
 
for rep in "${reps[@]}"; do

input_bam="${sample}_${stage}_${rep}.sorted_PE.sorted_DS.bam"

 
 
suffix="${sample}_${stage}_${rep}"

 
 
output_file="${output_dir}/${suffix}_DoGs_rpkm.txt"

 
 
dog_file="${union_dog_file}${sample}.bed"

 
 
Get_DoGs_rpkm -out "$output_dir" ∖

 
 
 
 
-bam "$input_bam" ∖

 
 
 
 
-suff "$suffix" ∖

 
 
 
 
-s ∖

 
 
 
 
-dog "$dog_file" ∖

 
 
 
 
-g ./Get_DoGs/genome_file.txt

 
 
 
# Move output file to correct location and rename it

 
 
 
mv "${output_dir}/${suffix}_DoGs_rpkm.txt" "$output_file"

 
 
 
done

 
done

done

# This R code refers to metagene plot for dRPB3 in Figure 4

# Note: similar code can be employed for RPB3.

# Load required packages

library(ggplot2)

library(reshape2)

library(ggpubr)

library(readxl)

# Read the Excel data into a dataframe, removing rows with missing values

df <- na.omit(read_excel("protocol_stats.xlsx", sheet = 3, trim_ws = TRUE, range = cell_cols(c("A","D"))))

# Display the first few rows of the dataframe

head(df)

# Convert the "sample" column to a factor with specified levels

df$sample <- factor(df$sample, levels = df$sample)

# Create a bar plot using ggbarplot from ggpubr

p3 <- ggbarplot(df, x = "sample", y = "count",

 
 
 
 
fill = "cond", color = "cond", palette = "npg",

 
 
 
 
label = TRUE,

 
 
 
 
position = position_dodge(0.8)) +

 
 
ggprism::theme_prism() +

 
 
theme(axis.text.x = element_text(angle = 40, hjust = 1))

# Save the plot as a PDF file

ggsave(p3, filename = "Figure 4_ Readthrough_gene_number.pdf", width = 10, height = 6)

# Create a PDF device for the Venn diagram

pdf("Figure 4_Venn_Diagram.pdf", 8, 8)

# Set up the plotting layout

par(mfrow = c(1, 2), bty = 'n')

# Draw the Venn diagram for dRPB3 using the total number of readthrough gene candidates from the bar plot, as well as determine their overlaps

venn.dRPB3 <- draw.triple.venn(area1 = 10065,

 
 
 
 
 
 
 
 
area2 = 10698,

 
 
 
 
 
 
 
 
area3 = 10703,

 
 
 
 
 
 
 
 
euler.d = TRUE, scaled = TRUE,

 
 
 
 
 
 
 
 
n12 = 9552 + 215, n23 = 9552 + 598, n13 = 9552 + 155, n123 = 9552,

 
 
 
 
 
 
 
 
category = c("IAA_0h", "IAA_1h", "IAA_3h"),

 
 
 
 
 
 
 
 
lty = "blank",

 
 
 
 
 
 
 
 
fill = c("#CF4C35", "#4DA8BE", "#1A937D"),

 
 
 
 
 
 
 
 
alpha = 0.5,

 
 
 
 
 
 
 
 
cex = 2, cat.cex = 2, cat.pos = c(-1, -1, 180))

# Draw the Venn diagram for RPB3

venn.RPB3 <- draw.triple.venn(area1 = 7195,

 
 
 
 
 
 
 
 
area2 = 7733,

 
 
 
 
 
 
 
 
area3 = 8024,

 
 
 
 
 
 
 
 
euler.d = TRUE, scaled = TRUE,

 
 
 
 
 
 
 
 
n12 = 6651 + 258, n23 = 6651 + 592, n13 = 6651 + 136, n123 = 6651,

 
 
 
 
 
 
 
 
category = c("IAA_0h", "IAA_1h", "IAA_3h"),

 
 
 
 
 
 
 
 
lty = "blank",

 
 
 
 
 
 
 
 
fill = c("#CF4C35", "#4DA8BE", "#1A937D"),

 
 
 
 
 
 
 
 
alpha = 0.5,

 
 
 
 
 
 
 
 
cex = 2, cat.cex = 2, cat.pos = c(-1, -1, 180))

# Close the PDF device

dev.off()

### Determine readthrough and non-readthrough genes

**Timing: ∼1 h**

Define readthrough genes.

36. Exclude very short genes with a length of less than 2 kb from the analysis.
37. Generate a sub-list of active genes from our untreated PRO-seq data.

***Note:*** Gene activity was determined based on the ratio of N/L, where N corresponds to the count of coding-strand Pro-seq reads from +1kb (relative to the TSS) to the end of each gene, and L denotes the number of mappable bases in this region. The statistical significance of a given gene's activity level was estimated by calculating the probability of observing a minimum of N reads within an interval of length L, derived from a Poisson distribution of mean λ = 0.04 reads/kb. Genes with a probability of less than 0.01 were defined as active, as described in our publication.^2^

38. Refine readthrough gene candidates identified in **step** 34 that overlap with active genes but do not overlap with readthrough regions that correspond to neighboring genes on either strand using Bedtools intersect analysis.
  a. Identify readthrough gene candidates that overlap with active genes.
  b. Find transcripts whose TSS or TTS region extended in 5′ and 3′ overlaps with any transcript from another gene.
  c. Remove readthrough gene candidates that overlap with neighboring genes on either strand.
  d. Keep each readthrough gene a unique name.

#!/bin/bash

# Note: Step 38is standalone and function independently.

# Variables are defined within this script.

# Define input files and directories

promoter_size=1000

# Find genes that overlap promoters

gtftk overlapping -i ./gencode_mm10_vM23/loci_annotation.bed -c mm10.chromInfo -t promoter -u $promoter_size -d $promoter_size | gtftk select_by_key -k feature -v transcript | gtftk tabulate -k gene_id,overlap_promoter_u${promoter_size}_d${promoter_size} > dist_to_overlapping_mm10.txt

# Use the paste command to join the files by columns

paste gencode.vM23.gene.bed dist_to_overlapping_mm10.txt ∖

  | awk -v FS='∖t' -v OFS='∖t' '{print $5,$1,$2,$3,$4,$6}' ∖

  | sort -k1,1 ∖

  > overlapping_gene.bed

# Use an array to store sample names

samples=(dRPB3_IAA RPB3_IAA)

# Use a for loop to iterate over the sample names

for sample in "${samples[@]}"; do

 bedtools intersect -s -a ./Final_UnionDog_annotation/Final_UnionDog_annotation_${sample}.bed -b gencode.vM23_active.bed ∖

  | sort -u > Final_UnionDog_annotation_active_${sample}.bed

 bedtools intersect -s same -v -a Final_UnionDog_annotation_active_${sample}.bed -b overlapping_gene.bed ∖

  | sort -u > ${sample}_readthrough.bed

# Use a single awk command to filter for the longest transcript per gene with unique name

 awk '{key=$4; if ($3-$2 > max[key]) {max[key]=$3-$2; row[key]=$0}} END{for (k in row) print row[k]}' ${sample}_readthrough.bed ∖

  | sort -k4,4 -u ∖

  > Final_${sample}_readthrough.bed

Done

***Note:*** The bedtools and gtftk tools have already been installed in the system's PATH.

Define non-readthrough genes.

39. Obtain the genomic coordinates for the active genes and readthrough regions of neighboring genes.
40. Identify overlap between the active genes and the readthrough regions according to bedtools intersect analysis.
  a. Create a list of active genes that do not overlap with readthrough regions of neighboring genes on either strand.
  b. Subtract the readthrough genes from this list of active genes to create a new list of non-readthrough genes that fail to generate readthrough transcripts.
  c. Ensure that each non-readthrough is given a unique name.

***Note:*** The script in this part is similar to that of defining readthrough genes.

### Quantify the readthrough indices

**Timing: ∼1–2 h**

41. Consider genes with a minimum length of 2 kb to exclude very short genes from the analysis. Troubleshooting 4.
42. Define two sub-genic windows as the following: termination windows are from 3 to 6 kb downstream of polyA sites; pre-polyA windows are 1 kb upstream of respective polyA sites.
43. Calculate the ChAR-seq read counts in two sub-genic windows.
44. Filter out genes with fewer than five reads in either termination or pre-polyA windows from the analysis.
45. Determine the readthrough index as the ratio of the ChAR-seq read coverage in the termination window to that in the pre-polyA window.

***Optional:*** Build high-quality 3′UTR polyadenylation sites (PASs) for mouse genome directly from GENCODE annotation GTF files (M23). It may be helpful to refine genes that contain polyadenylation sites.

# This R code refers to an option of step 45

% if (!"BiocManager" %in% rownames(installed.packages()))

install.packages("BiocManager")

BiocManager::install("APAlyzer")

# Load required packages

library(APAlyzer)

GTFfile="gencode.vM23.annotation.gtf.gz"

PASREFraw=PAS2GEF(GTFfile)

refUTRraw=PASREFraw$refUTRraw

UTRdbraw=REF3UTR(refUTRraw)

write.table(UTRdbraw, "UTRdbraw.tsv", append = TRUE, sep = "∖t")

***Note:*** our quantification pipeline includes a custom Perl (Calculate_readthrough_indices.pl) that (i) use downsampled reads from **step** 32 and the bed file from **step** 45 to divide read coverages in pre-polyA window by termination window to generate readthrough indices and (ii) make sure genes are at least 2 kb long.

#!/usr/bin/perl

use strict;

use warnings;

use File::Basename;

use File::Spec::Functions qw(catfile);

# Get command line arguments

my $genes_file = $ARGV[0];

my $frags_file = $ARGV[1];

# Check if files exist

die "Error: Genes file $genes_file not found∖n" unless -e $genes_file;

die "Error: Fragments file $frags_file not found∖n" unless -e $frags_file;

# Define output file names

my $genes_over_2kb_file = 'genes.over2kb.bed';

my $term_window_file = 'term.bed';

my $poly_window_file = 'poly.bed';

my $term_sorted_file = 'term.srt.bed';

my $poly_sorted_file = 'poly.srt.bed';

my $term_count_file = 'term.count';

my $poly_count_file = 'poly.count';

# Make sure genes are at least 2 kb long

system("awk '{if (∖$10-∖$9>2000) print ∖$0}' $genes_file > $genes_over_2kb_file");

print "Genes < 2000bp parsed out∖n∖n";

# Define new termination window from TTS+3000 to TTS+6000 (only genes >= 2000 bp and not overlapping any other gene)

open $over2kb_fh, '<', $genes_over_2kb_file or die "Cannot open $genes_over_2kb_file: $!";

open my $term_fh, '>', $term_window_file or die "Cannot open $term_window_file: $!";

while (my $line = <$over2kb_fh>) {

 
chomp $line;

 
my @fields = split(/∖t/, $line);

 
if ($fields[5] eq '+') {

 
 
print $term_fh join("∖t", $fields[0], $fields[2] + 3000,

$fields[2] + 6000, @fields[3..5]), "∖n";

 
} elsif ($fields[5] eq '-') {

 
 
print $term_fh join("∖t", $fields[0], $fields[1] - 6000,

$fields[1] - 3000, @fields[3..5]), "∖n";

 
}

}

close $over2kb_fh;

close $term_fh;

# Define new pre-polyA window from TTS-1000 to TTS+0 (only genes >= 2000 bp and not overlapping any other gene)

open $over2kb_fh, '<', $genes_over_2kb_file or die "Cannot open $genes_over_2kb_file: $!";

open my $poly_fh, '>', $poly_window_file or die "Cannot open $poly_window_file: $!";

while (my $line = <$over2kb_fh>) {

 
chomp $line;

 
my @fields = split(/∖t/, $line);

 
if ($fields[5] eq '+') {

 
 
print $poly_fh join("∖t", $fields[0], $fields[2] - 1000, $fields[2], @fields[3..5]), "∖n";

 
} elsif ($fields[5] eq '-') {

 
 
print $poly_fh join("∖t", $fields[0], $fields[1], $fields[1] + 1000, @fields[3..5]), "∖n";

 
}

}

close $over2kb_fh;

close $poly_fh;

# Sort termination and gene polyA window files

system("sort -k1,1 -k2,2n $term_window_file > $term_sorted_file");

system("sort -k1,1 -k2,2n $poly_window_file > $poly_sorted_file");

print "Windowed files sorted∖n∖n";

# Calculate coverage over windows, run bedtools with -s option for strandedness (ChAR-seq)

system("bedtools coverage -s -a $term_sorted_file -b $frags_file -counts > $term_count_file") == 0

 
or die "bedtools coverage failed for $term_sorted_file∖n";

system("bedtools coverage -s -a $poly_sorted_file -b $frags_file -counts > $poly_count_file") == 0

 
or die "bedtools coverage failed for $poly_sorted_file∖n";

print "Read coverage over windows calculated∖n∖n";

# Open coverage files

open my $promcov, "<", "term.count" or die "Can't open term.count: $!";

open my $genecov, "<", "poly.count" or die "Can't open poly.count: $!";

open my $bed, ">", "readthrough_indices.txt" or die "Can't open readthrough_indices.txt: $!";

# Print header to output file

print $bed "poly_chr∖tpoly_start∖tpoly_stop∖tpoly_strand∖tpoly_name∖tpoly_cov∖tterm_name∖tterm_start∖tterm_stop∖tterm_strand∖tterm_cov∖tri∖n";

# Read through coverage files

my %poly_cov;

while (my $line1 = <$promcov>) {

 
chomp $line1;

 
my ($chr, $start, $stop, $name, $cigar, $strand, $cov) = split /∖t/, $line1;

 
$poly_cov{$name} = "$chr&$start&$stop&$name&$cigar&$strand&$cov";

}

while (my $line2 = <$genecov>) {

 
chomp $line2;

 
my ($term_chr, $term_start, $term_stop, $term_name, $term_cigar, $term_strand, $term_cov) = split /∖t/, $line2;

# Check if termination coverage file has matching name to polyA window coverage file

 
if (exists $poly_cov{$term_name}) {

 
 
my $storedvalue = $poly_cov{$term_name};

 
 
my ($poly_chr, $poly_start, $poly_stop, $poly_name, $poly_cigar, $poly_strand, $poly_cov) = split /&/, $storedvalue;

 
 
# Calculate readthrough indices

 
 
if ($poly_cov > 5 && $term_cov > 5) {

 
 
 
my $poly_density = $poly_cov / 3000;

 
 
 
my $term_size = $term_stop - $term_start;

 
 
 
my $term_density = $term_cov / 1000;

 
 
 
my $ri = $poly_density / $term_density;

 
 
 
print $bed "$poly_chr∖t$poly_start∖t$poly_stop∖t$poly_strand∖t$poly_name∖t$poly_cov∖t$term_name∖t$term_start∖t$term_stop∖t$term_strand∖t$term_cov∖t$ri∖n";

 
 
 
}

 
}

}

# Close files

close $promcov;

close $genecov;

close $bed;

exit;

46. Compute readthrough indices of readthrough genes and non-readthrough genes for every bam file in **step** 32**.**

#!/bin/bash

# Note: Step 46 is standalone and function independently.

# Variables are defined within this script.

# Loop through samples, stages, and genes to calculate readthrough indices

for sample in dRPB3_IAA RPB3_IAA; do

 
for stage in 0h 1h 3h; do

 
 
for gene in readthrough non_readthrough; do

 
 
 
BED_FILE="${sample}_${stage}_${gene}.bed"

BAM_FILE="${sample}_${stage}_merged.sorted_PE.sorted_DS.bam"

 
 
 
OUT_FILE="${sample}_${stage}_${gene}.txt"

 
 
 
# Run Calculate_readthrough_indices.pl command

 
 
 
perl Calculate_readthrough_indices.pl "$BED_FILE" "$BAM_FILE" > "$OUT_FILE" || { echo "Calculate_readthrough_indices.pl failed for $BED_FILE and $BAM_FILE"; exit 1; }

 
 
 
done

 
done

done

### Compare the changes of 3′end processing upon RPB3 and dRPB3 depletion

**Timing: ∼30 min**

47. Apply the DESeq2 package to perform the differential 3′end processing analyses before (IAA-0 h) and after (IAA-3 h) dRPB3 or RPB3 depletion.

***Note:*** The readthrough indices are float numbers generated in **step** 45, which need to be rounded into an integer.

**Figure5:**
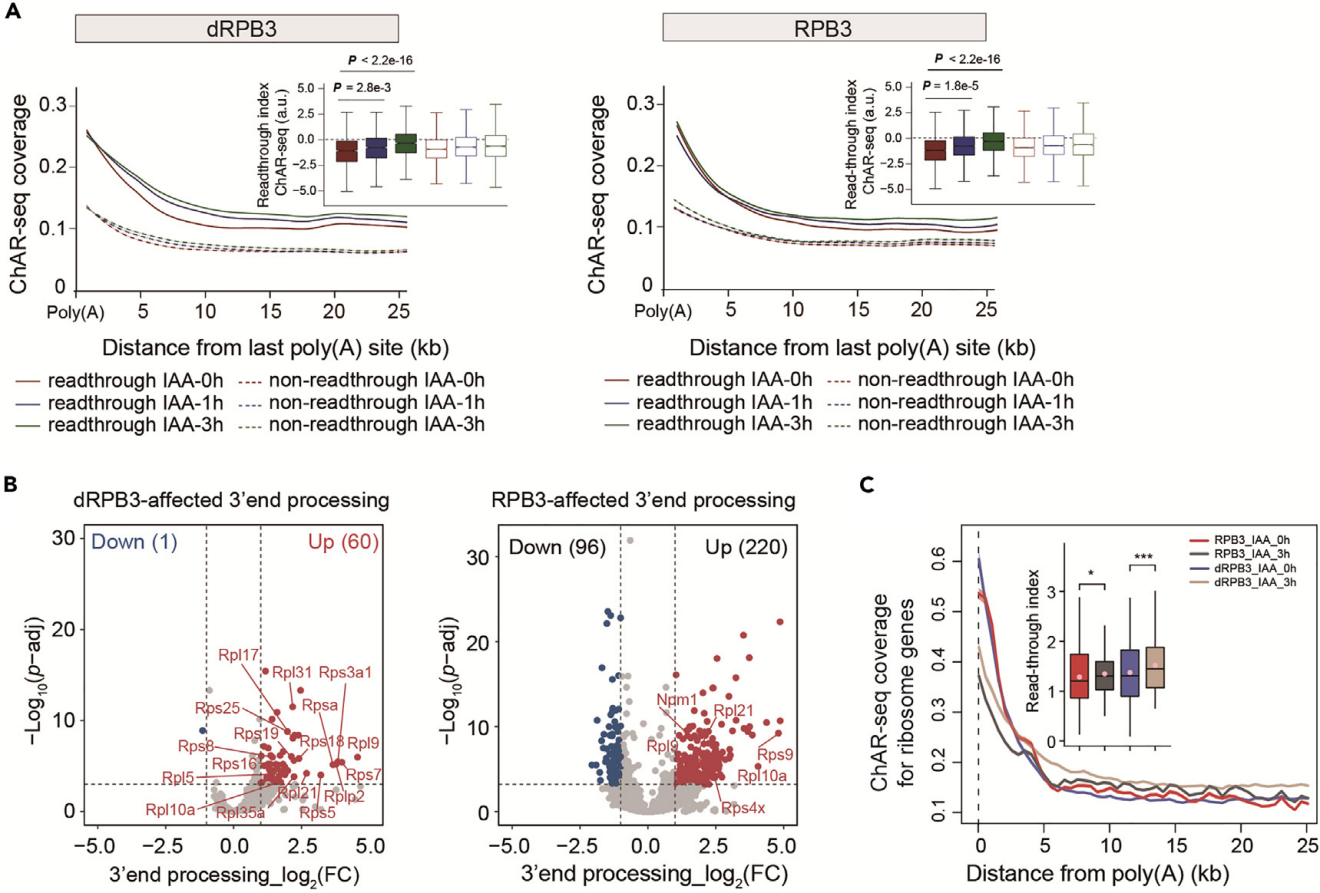
Results of ChAR-seq analysis (A) Metagene profiles of ChAR-seq signals at the polyadenylation sites of readthrough and nonreadthrough transcripts at different time points (0 h, 1 h, and 3 h) after IAA treatment in dRPB3 (left) or RPB3 (right) degron cells. Boxplots showing ChAR-seq readthrough indices of gene sets identified by DoGFinder, presented as the median in an interquartile range with whiskers indicating 1.5-fold the interquartile range and confidence region notches. A two-sided Wilcoxon rank-sum test was performed to determine significance. The data were reported as the average of two biological replicates for each treatment. (B) Volcano plots of induced 3′ end-processing changes upon dRPB3 (left) or RPB3 (right) depletion, as determined by ChAR-seq. (C) Metagene analysis of spike-in–normalized ChAR-seq reads around annotated polyadenylation sites of ribosomal subunit genes before and after depletion of RPB3 or dRPB3. The corresponding 3′ end RNA processing changes were quantified and displayed as boxplots. The middle line, lower hinge and upper hinge in the boxplot indicate the median, first quartile and third quartile, respectively. Significance was determined by a two-tailed Wilcoxon rank-sum test. ∗P < 0.05, ∗∗∗P < 0.001. Figure reprinted and reorganized with permission from Li et al.^1^

48. Organize the readthrough indices data into the matrix format required for DESeq2 analysis.
49. Select genes whose adjusted p-values are less than 0.05 and whose absolute fold changes are greater than 2 (Figure 5B).

# This chunk of R code refers to steps 47 and 49

# Load required packages

library(DESeq2)

library(org.Mm.eg.db)

library(data.table)

# read data

rawcount <- data.table::fread("dRPB3_combined_RIs.tsv")

# select columns of interest and set GeneID as row names

ct <- data.frame(rawcount[, c("dRPB3_IAA_0h_rep1", "dRPB3_IAA_0h_rep2", "dRPB3_IAA_3h_rep1", "dRPB3_IAA_3h_rep2")])

row.names(ct) <- rawcount$GeneID

# create column data

colData <- data.frame(

 
condition = factor(rep(c("0h", "3h"), each = 2)),

 
ind = factor(c(1, 2, 1, 2)),

 
row.names = colnames(ct)

)

# create DESeqDataSet object

dds <- DESeqDataSetFromMatrix(

 
countData = round(ct, 0),

 
colData = colData,

 
design = ∼ condition + ind

)

# filter genes with no counts

dds <- dds[rowSums(counts(dds)) > 0, ]

# estimate size factors

dds <- estimateSizeFactors(dds)

# run DESeq2

dds <- DESeq(dds)

res <- results(dds, contrast = c("condition", "3h", "0h"))

summary(res)

# create differential expression table

diff <- data.frame(res@listData, row.names = res@rownames)

lfc_thresh <- 1

padj_thresh <- 0.05

# classify genes based on log2FoldChange and adjusted p-value

diff$reg <- "unchanged"

diff$reg[diff$log2FoldChange < -lfc_thresh & diff$padj < padj_thresh] <- "down"

diff$reg[diff$log2FoldChange > lfc_thresh & diff$padj < padj_thresh] <- "up"

# display the number of genes in each category

table(diff$reg)

diff$ensembl_id <- rownames(diff)

# Mapping Ensembl IDs to gene symbols using org.Mm.eg.db

gene_name <- mapIds(x=org.Mm.eg.db, keys=diff$ensembl_id, column = "SYMBOL", keytype = "ENSEMBL")

gene_name <- data.frame(ensembl_id=names(gene_name), gene=gene_name)

# Joining 'gene_name' and 'diff' data frames

diff <- plyr::join(gene_name, diff, type="full")

head(diff)

# Creating a data frame of normalized counts

norm_ct <- as.data.frame(counts(dds, normalized = T))

norm_ct$ensembl_id <- rownames(norm_ct)

# Joining 'norm_ct' and 'diff' data frames

diff <- plyr::join(norm_ct, diff, type="right")

head(diff)

this_tile <- paste0('Up : ',nrow(diff[diff$reg =='up',]) ,' | ','Down : ',nrow(diff[diff$reg =='down',]),' | ','Unchange : ',nrow(diff[diff$reg =='unchanged',]))

# Calculating the number of genes in each category

ndown <- table(diff$reg)[1]

nunchange <- table(diff$reg)[2]

nup <- table(diff$reg)[3]

# Creating a scatterplot for dRPB3 as displayed inFigure 5B

# Note: similar code can be employed for RPB3.

p4 <- ggplot(data = diff, aes(x = log2FoldChange, y = -log(padj), color = reg)) +

geom_point() +

 
ggrepel::geom_text_repel(data = filter(diff, gene %in% label_ribo), aes(label = gene), box.padding = 0.5, max.overlaps = Inf) +

 
geom_hline(yintercept = -log(0.05), linetype = "dashed", color = "grey30") +

 
geom_vline(xintercept = -1, linetype = "dashed", color = "grey30") +

 
geom_vline(xintercept = 1, linetype = "dashed", color = "grey30") +

 
xlab("log2 (FC)") +

 
ylab("-log10 (p-adj)") +

 
ggtitle("R3F 3h-VS-Dox Readthrough Index") +

 
scale_x_continuous(limits = c(-5, 5)) +

 
scale_y_continuous(limits = c(0, 30)) +

 
scale_color_manual(values = c("#005792", "grey", "#ca3e47"), name = "",

 
 
 
 
 
 
label = c(paste0("down (", ndown, ")"), paste0("unchanged (", nunchange, ")"), paste0("up (", nup, ")"))) +

 
theme(plot.title = element_text(colour = "black", size = 20, hjust = 0.5),

 
 
 
axis.line.x = element_blank(), axis.line.y = element_blank(),

 
 
 
legend.text = element_text(size = 15),

 
 
 
axis.text = element_text(colour = "black", size = 17), axis.title = element_text(size = 20),

 
 
 
panel.border = element_rect(colour = "black", fill = NA, size = 0.5),

 
 
 
panel.background = element_blank())

# Saving the scatterplot as a PDF file

ggsave(p4, filename = "Figure 5B_dRPB3_scatterplot.pdf", width = 5, height = 4)

50. Conduct GO term analysis to show distinct gene ontologies of 3′end processing-affected genes linked to ribosomal subunit genes, as described in our publication.^1^
51. Perform a Wilcoxon test to assess the statistical significance of differences in 3′end processing before and after dRPB3 or RPB3 depletion (Figure 5C).

## Expected outcomes

We performed ChAR-seq data analysis on six samples following this protocol. Our findings indicate that dRPB3 regulates the 3′ end processing of ribosomal subunit genes. Specifically, the results demonstrate that the detected readthrough genes in dRPB3 were significantly higher than those in RPB3 degron cells (Figure 5A). Moreover, Differential 3′ end processing analysis between samples suggests that dRPB3 plays an important role in RNA processing, given the higher proportion of observed changes in the readthrough index for ribosomal subunits upon dRPB3 depletion (Figure 5B). Finally, in line with prior findings, metagene analysis of ChAR-seq signals at TTS revealed increased transcriptional readthrough for ribosomal subunit genes after dRPB3 depletion (Figure 5C).

# This R code refers to metagene plot for dRPB3 inFigure 5A

# Note: similar code can be employed for RPB3.

# Load required packages

library(ggplot2)

library(reshape2)

library(ggpubr)

# Reading data from a file and storing it in 'df' data frame

df <- read.delim("dRPB3_metaTTS_bin50_ChARseq.tab", header = TRUE)

# Reshaping the data frame using melt function from reshape2 package

df_long <- reshape2::melt(df, id = c("group", "type", "class"))

# Removing "X" from the variable names

df_long$variable <- gsub("[X]", "", df_long$variable)

# Rearranging the data frame by 'group' column and updating the factor levels of 'variable'

df_long <- df_long %>%

 arrange(group) %>%

 mutate(variable = factor(variable, levels = unique(df_long$variable)))

# Creating line plots using ggplot2

ggplot(data = df_long, aes(x = variable, y = value, colour = type, fill = type, group = type)) +

 geom_smooth(method = "loess", span = 0.3, alpha = 0.4) +

 facet_wrap(group ∼ class, ncol = 2) +

 scale_fill_manual(values = c("darkred", "darkblue", "darkgreen")) +

 scale_color_manual(values = c("darkred", "darkblue", "darkgreen")) +

theme_pubr()

# Saving the plot to the PDF file

ggsave(p5, filename = "Figure 5A_dRPB3_metagene.pdf", width = 5, height = 4)

# This R code refers to box plot for dRPB3 inFigure 5A

# Note: similar code can be employed for RPB3.

# Reading data from files and selecting specific columns

df_x <- read.delim("dRPB3_readthrough_RIs_combine.tsv", header = TRUE)[, 4:8]

df_y <- read.delim("dRPB3_nonreadthrough_RIs_combine.tsv", header = TRUE)[, 4:8]

# Reshaping the data frames using melt function from reshape2 package

df_x <- reshape2::melt(df_x, id = c("group", "gene"))

df_y <- reshape2::melt(df_y, id = c("group", "gene"))

# Taking the log2 of 'value' columns in both data frames

df_x$value <- log2(df_x$value)

df_y$value <- log2(df_y$value)

# Combining the reshaped data frames

df <- rbind(df_x, df_y)

# Creating a grouped box plot using ggboxplot from ggpubr

p6 <- ggboxplot(df, x = "variable", y = "value",

     fill = "variable", palette = c("#841a1f", "#103888", "#086233", "#841a1f", "#103888", "#086233"),

     bxp.errorbar = TRUE, notch = TRUE, outlier.shape = NA,

     add.params = list(position = position_jitter(0.8), size = 3), shape = "variable", facet.by = "group") +

     ylim(-6, 4.5) + ggpubr::theme_pubr()

# Specifying comparisons for statistical significance testing

my_comparisons <- list(c("IAA_0h", "IAA_1h"), c("IAA_0h", "IAA_3h"))

# Adding statistical comparisons and labels to the plot

p6 <- p6 + stat_compare_means(comparisons = my_comparisons) + stat_compare_means(label.y = 2)

# Saving the plot as a PDF file

ggsave(p6, filename = "Figure 5A_dRPB3_boxplot.pdf", width = 5, height = 4)

# Line and box plots to display the 3' end RNA processing changes for ribosomal subunit genes as inFigure 5C

# Read the data from a file and store it in the 'df' data frame

df <- read.delim("ribosome_metaTTS_bin50_ChARseq.tab", header = TRUE)

# Reshape the data frame using the melt function from the reshape2 package

df_long <- reshape2::melt(df, id = c("group", "type"))

# Remove "X" from the variable names

df_long$variable <- gsub("[X]", "", df_long$variable)

# Rearrange the data frame by the 'group' column and update the factor levels of 'variable'

df_long <- df_long %>%

 arrange(group) %>%

 mutate(variable = factor(variable, levels = unique(df_long$variable)))

# Create line plots using ggplot2

p7 <- ggplot(data = df_long, aes(x = variable, y = value, colour = type, fill = type, group = type)) +

 geom_line() +

 scale_fill_manual(values = c("#D83232", "#595959", "#5261AA", "#C7B299")) +

 scale_color_manual(values = c("#D83232", "#595959", "#5261AA", "#C7B299")) + ggpubr::theme_pubr()

# Save the plot to a PDF file

ggsave(p7, filename = "Figure 5C_ribosome_metagene.pdf", width = 5, height = 4)

# Read in the data and reshape it using melt from the reshape package

ribosome_RI <- read.csv("ribosome_RIs_combine.tsv", header = TRUE)

ribosome_RI <- reshape::melt(ribosome_RI, id.vars = "gene")

# Define the comparisons to be made in the plot

my_comparisons <- list(c("dRPB3_IAA_0h", "dRPB3_IAA_3h"), c("RPB3_IAA_0h", "RPB3_IAA_3h"))

# Create a grouped box plot using ggboxplot from ggpubr

p8 <-ggpubr::ggboxplot(data = ribosome_RI, x = "variable", y = "value",

      fill = "variable", outlier.shape = NA,

      palette = c("#D83232", "#595959", "#5261AA","#C7B299"),

      yscale = "log10", add = "mean", add.params = list(color = "pink")) +

  ggpubr::stat_compare_means(comparisons = my_comparisons, label = "p.signif") +

  ggpubr::stat_compare_means(label.y = 2) +

  ggpubr::theme_pubr()

# Save the plot as a PDF file

ggsave(p8, filename = "Figure 5C_ribosome_boxplot.pdf", width = 4, height = 6)

## Quantification and statistical analysis

For the differential 3′ end processing analysis, the changes in the readthrough index were identified using the negative binomial generalized linear models implemented in the DESeq2 R package. Statistical significance was assessed by a two-sided Wilcoxon rank-sum test.

## Limitations

The Specific Degradation of Disassociated Subunits (SDDS) strategy employed in our study could be extended to investigate the disassociated subunits of other multi-molecular complexes such as ribosomes, spliceosomes, and DNA polymerases. However, to apply the SDDS approach to investigate such complexes, it is necessary to optimize the design to ensure that fusions with specific interacting proteins faithfully mimic the functions of endogenous protein complexes and do not cause noticeable adverse effects. In addition, deletion mutants, combined with functional assays, would be ideal to further validate the conclusions drawn from the experimental results.

To investigate the immediate functions of dRPB3 and RPB3 in RNA processing, we established a degron system for these proteins in murine embryonic stem cells and utilized auxin-inducible degron (AID) technology^1^^,^^2^^,^^14^^,^^15^^,^^16^^,^^17^ to rapidly deplete them. We then conducted chromatin-associated RNA experiments with spike-in controls in Drosophila S2 cells to evaluate the impact of their depletion. This approach is similar to that employed in previous studies.^18^^,^^19^ To enhance the statistical confidence of the ChAR-seq data analysis, we included two biological replicates in all the datasets to minimize experimental bias and ensure the robustness of the statistics performed using this protocol. For example, the replicates were treated separately for significance assessment for differential expression and 3′ end processing analysis, whereas for constructing metagene profiles, replicates were merged to maximize the power of revealing differences in the gene body and readthrough signals. Nonetheless, this protocol did not test datasets with a variable number of replicates, leaving open the potential issue of how to process such data. Therefore, the handling of replicates should be tailored to each experiment stage and analysis purpose to ensure the reliability of the results.

## Troubleshooting

### Problem 1

Low yield of RNA extraction of chromatin-associated RNA may arise in **step 8**.

### Potential solution

• Increase the starting materials (e.g., larger number of cells).
• Adjust the amount of TRIzol (for RNA extraction) to avoid incomplete cell lysis due to the insufficient amount of TRIzol.
• Prolonged lysis with TRIzol reagent can be applied to fully resuspend the viscous and sticky chromatin pellet. It can be fully resuspended by repeatedly pipetting or vortexing until no chromatin pellet can be observed.

### Problem 2

Low mapping reads of dm6 may occur in **step 17**.

### Potential solution

• Increase the percentage of spike-in Drosophila S2 cells.

### Problem 3

Compatibility issues due to old versions of software and algorithms used in the protocol.

### Potential solution

• We mentioned this issue in the Note of the software and algorithms section. In brief, all the custom scripts run seamlessly with the R version (v.4.1.1), Perl (v5.26.2) and Python (v3.7.4), and the same results can be reproduced. Although we anticipate that forthcoming versions of software employed for NGS read processing will sustain their analytical and statistical efficacy using the same basic parameters, their performance on our data has yet to be evaluated. In the near future, we aim to provide a Docker image containing the identical software version on the Github repository to resolve the issue.

### Problem 4

When running a piece of code, the output is not the expected (i.e., an error message in the console or an unexpected feature in the plot).

### Potential solution

• For the proper execution of the piece of code, it is important to verify that the syntax is correct. Specifically, examine for syntax errors, such as missing commas, unclosed brackets or parentheses, quotations, and misspelled functions or filenames.

### Problem 5

The variability in the number of readthrough gene candidates identified by DoGfinder in step 32, depends on the sequencing and library type.

### Potential solution

• Adjust the parameters used in the readthrough gene identification process, given its reliance on continuous coverage criteria. Specifically, stricter minimal length and coverage parameters could be chosen when analyzing non-polyA selected RNA-seq libraries, since readthrough genes are known to remain nuclear. In our recent work,^2^ we have adopted default parameters of 4000 bases and 60% coverage for polyA-selected RNA-seq libraries, which have been found to be appropriate in previous studies.^12^^,^^20^
• To further refine the list of candidates, we suggest considering highly expressed genes by limiting the selection to those exceeding a certain RPKM value threshold.

### Problem 6

Batch effects may exist among different samples.

### Potential solution

• Perform sample clustering and utilize visualization tools, including PCA plots and scatterplots, to comprehensively evaluate batch effects. For experiments involving different batches, it is recommended to apply specialized software such as SVA or RUVseq to correct batch effects accurately.

## Resource availability

### Lead contact

Further information and requests for resources and reagents should be directed to the lead contact, Xiong Ji (xiongji@pku.edu.cn).

### Materials availability

Regents generated in this study are available upon request to thelead contact.

## Data Availability

The data used in this study are described in Li et al. (2023).^1^ The GEO number of RPB3 ChAR-seq data is GEO: GSE179962. The GEO number of dRPB3 ChAR-seq data is GEO: GSE225453. This protocol includes all codes. The source code for executing this pipeline is readily available for download as a supplementary file and archived at Zenodo [https://doi.org/10.5281/zenodo.7936207].

## Supplemental information

Data S1. Scripts for ChAR-seq analysis, related to steps 1–16
